# Hospital Annual Meetings

**Published:** 1919-04-19

**Authors:** 


### HOSPITAL ANNUAL MEETINGS.

### London Homœopathic Hospital.

At the seventieth annual meeting of the hospital in Great Ormond Street, Lord Donoughmore in the chair, it was reported the weekly cost per bed had increased from £1 14s. in 1914 to £2 4s. 2d. in 1918. Throughout the war eighty-five beds had been occupied with naval casualties, 1,800 sailors having received surgical or medical treatment. Sir William Norman, Director-General of Medical Services at the Admiralty, wrote: "I very much regret that owing to a previous engagement I shall not be able to attend your annual meeting. Will you please be good enough to express my thanks to the Governors of the Hospital for the care and treatment bestowed on sick and wounded officers and men of the Royal Navy whom they have had under their charge during the period of the war. On the conclusion of peace negotiations I am forwarding the Lords Commissioners of the Admiralty a report of the good work done by the medical and nursing staff of your hospital, pointing out how much their services have been valued and appreciated by the numerous patients that have been under treatment, and I feel sure that their Lordships will forward you a letter of recognition and thanks for the great work done by this hospital." The Board have recently invited ladies to join their number, and the Countess of Donoughmore, Lady Perks, and Mrs. Alex. Balfour Williamson have accepted seats on the Board of Management. There was an overdraft at the bankers on December 31 last of £1,294, which has since increased. To decrease this overdraft a lady has promised £100 if five others will do the same. The Earl of Dysart and Lady Durning Lawrence have each donated £100, and it is hoped the remaining three donors will be obtained.

### The Infants Hospital.

Lord Cheylesmore presided over the annual Court of Governors at Vincent Square. Last year 349 in-patients were cared for, of whom 107 have made good recoveries. New out-patients numbered 859 with 3,067 attendances. The total indebtedness of the hospital has been increased to £3,080 by the addition of a deficit of £356 on last year's working.

### The Cancer Hospital.

The Earl of Northbrook presided over the annual meeting at the Hospital, Fulham Road, which last year cared for 721 in-patients, as compared with 636 in 1917. New out-patients numbered 1,075 and their attendances 20,310, against 1,225 and 29,350 a year previously. During 1918 211 officers and men have been treated in the electrical department. Two of the wards have been closed during the year under review, owing to the shortage of nurses. National War Bonds to the value of £2,000 have been presented to the funds of the Charity by the executors of the late H. F. Bailey, while the committee have received from the late Mrs. Tupper a legacy of £3,000 in memory of her son, Lieut. Charles Tupper, R.N. The work of the electrical and radio-therapeutic department is progressing, over 13,000 attendances of patients being recorded last year.

### St. Peter's Hospital.

At the annual meeting at the hospital, Covent Garden, under the presidency of Mr. Edwin Fox, it was reported that during the past year 380 patients had been admitted to the wards, which now contained thirty-two beds. All but twenty were cured or relieved. Out-patients treated numbered 2,372, and their attendances totalled 31,755. Income in 1918 was £7,474 and exceeded expenditure by £455. A bed has been endowed by the gift of £1,000 from the executors of the late Sir John Howard. Dr. J. E. Palmer has completed his term of office as house surgeon, and Mr. J. Morgan Hughes, M.R.C.S., has been appointed to the senior post and Major H. E. Cumming, M.C., to the junior office.

### St. Paul's Hospital.

Sir Thomas Dewar presided at the Holborn Restaurant over the annual meeting of St. Paul's Hospital, Red Lion Square, W.C., which last year cared for 626 in-patients—135 more than in 1917. New out-patients numbered 1,751, as compared with 1,945 a year previously. Their attendances, however, had increased by 3,641. Income had risen from £1,896 in 1917 to £2,870 last year. That figure included £912 contributed to the institution's Extension Fund. Expenditure, however, was £2,291 last year, as compared with £1,534 in 1917.

### Hospital for Throat Diseases.

Under the chairmanship of Mr. James Jackson, the annual general meeting of the hospital in Golden Square was held, when it was stated that last year in-patients numbered 1,067 as compared with 878 in 1917. In addition, 1,683 persons had been admitted for minor operations necessitating their retention for the day. New out-patients had increased from 8,985 with 39,737 attendances in 1917 to 10,717 with 46,365 attendances during the period under review. Subscriptions and donations in 1918 amounted to £4,645, as against £3,020 a year previously.

